# Near IR responsive targeted integrated lipid polymer nanoconstruct for enhanced magnolol cytotoxicity in breast cancer

**DOI:** 10.1038/s41598-020-65521-z

**Published:** 2020-05-29

**Authors:** Mona Elhabak, Rihab Osman, Mona Mohamed, Ola M. El-Borady, Gehanne A. S. Awad, Nahed Mortada

**Affiliations:** 1 Department of Pharmaceutics and Industrial Pharmacy, Faculty of Pharmacy, Ahram Canadian University, 6th of October, Egypt; 2 Department of Pharmaceutics and Industrial Pharmacy, Faculty of Pharmacy, Ain Shams University, Cairo, Egypt; 3 National Institute of Laser Enhanced Science, Cairo University, Cairo, Egypt; 4 Institute of Nanoscience and Nanotechnology, Kafrelsheikh University, Kafrelsheikh, Egypt

**Keywords:** Cancer, Nanoscience and technology

## Abstract

Advances in cancer nanotechnology aim at improving specificity and effectiveness for tumor treatment. Amalgamation of different treatment modalities is expected to provide better cancer combating. Herein, We developed a long circulating nanocarrier comprising trastuzumab (TZB) surface modified polylactic-*co*-glycolic acid (PLGA) nanoparticles (NPs) *co*-encapsulating magnolol (Mag) and gold nanoparticles (GNPs). A modified single step nanoprecipitation method was adopted ensuring particle coating with D-α-tocopheryl polyethylene glycol 1000 succinate (TPGS) while *co*-encapsulating GNPs. TZB was then anchored on NPs surface using a carbodiimide chemistry. The cytotoxicity of the developed system was evaluated with and without photothermal irradiation. NPs cellular uptake was then followed using confocal microscopical imaging. A hybrid matrix composed of PLGA/TPGS and surface decorated with TZB with a conjugation efficiency of ˃65%, was confirmed *via* FTIR, ^1^HNMR. GNPs could only be included in the NPs, when placed in the organic phase as evidenced by the shifted GNPs surface plasmonic resonance and confirmed *via* imaging coupled with energy dispersive X-ray analysis. Optimized NPs (136.1 ± 1.3 nm, −8.2 ± 1 mV and Mag encapsulation efficiency of 81.4 ± 1.8%) were able to boost Mag cytotoxicity on breast cancer cells while providing a selective multifunctional therapy with an added photothermal effect.

## Introduction

Despite the great advances in cancer research, breast cancer remains one of the most challenging diseases risking the lives of a considerable proportion of women world wide^1^. Lack of selectivity, poor drug penetration in solid tumors, multi drug resistance (MDR) with increased cells sensitivity added to the drug systemic side effects, represent some of the major barriers for a successful therapy^2^. Smart drug delivery systems incorporating different cancer targeting approaches including: passive, active and stimuli responsive had been suggested to improve outcomes of cancer treatment^3,4^. In this study, we focused on integrating many strategies that could potentiate the effect of a natural cytotoxic drug with potential activity against breast cancer.

Magnolol (Mag), see structure Fig. S1, is a phenolic polyhydroxy compound isolated from the root and stem bark of *Magnolia Officinalis*^5^. It had been found to inhibit proliferation and induce apoptosis in MCF-7 breast cancer cells *via* the intrinsic pathway with release of apoptosis inducing factor from mitochondria^5,6^. Studies have also shown that Mag inhibited cell growth and HER2- mediated tumor metastasis in human HER2- cancer cells^7^. Although, Mag might be considered a potential lead drug in breast cancer treatment yet, its delivery is usually hampered by poor aqueous solubility and low oral bioavailability^6,8^. Hence, its incorporation in a smart nanocarrier is expected to improve its delivery characteristics and boost its anticancer properties especially with the incorporation of multifunctional adjuvants.

Various types of targeting moieties have been employed to actively target NPs to cancer cells including; antibodies, antibody fragments, aptamers, transferrin and others^9^. The use of a certain targeting ligand depends on the prevalence of particular receptors on their surface. Breast carcinogenesis has been associated with overexpression of various cell membrane receptors including: human epidermal growth factor-2 (HER2), vascular endothelial growth factor, insulin-like growth factor I and hormone receptors; either progesterone or estrogen^10–12^. Though HER2 over-expression breast cancer type is known to be of very rapid cell proliferation, targeting these receptors have widely affected the quality of life of breast cancer patients^10^. Trastuzumab (TZB), also called herceptin, had been widely used for actively targeting breast cancer with large expression of HER-2^12–14^. For instance, TZB coated lipid-polymer hybrid nanoparticles (NPs) loaded with docetaxel showed increase in cell uptake depending on HER2 expression level^15^. In a comparative cell uptake study, TZB modified gold NPs exhibited higher affinity and cytotoxicity towards HER2-overexpressing human ovarian SKOV-3 cell line compared to non targeted NPs^16^. TZB modified NPs were effectively internalized and distributed near the nucleus within HER2-positive cancer cells^16^. Furthermore, *in vivo* studies revealed that TZB decorated liposome-PEG-PEI complex (LPPC) improved the site specific delivery of docetaxel-loaded LPPC to the tumor area but not to the healthy organs^17^. In addition, designing the NPs in the size range of 100–200 nm while applying a camouflaged surface coating, passive accumulation of these nanocarriers, resulting from their long circulation and enhanced permeation and retention, will synergize their active targeting^18^.

The potential of organic nanomaterials in the design of smart systems, which deliver their cargo in response to an external stimulus, have been widely acknowledged in breast cancer therapy^19–21^. Nevertheless, inorganic NPs made of a metal core (iron oxide, gold or quantum dots) have been also of wide interest in cancer therapy^22^. Gold NPs (GNPs), consisting either entirely or partially of gold, turn up to be ahead as a therapeutic platform because of several advantages. These include: a rich surface chemistry which has endowed their conjugation with various site specific molecules, light absorption properties appended their use for local tumor photothermal ablation^23^, passive tumor targeting boosting the action of the used anticancer with an elevated localized tumor cytotoxicity due to the gold photothermal effect^24,25^. An efficient adjuvant in breast cancer therapy where the use of gold can confine adequate thermal dosage to tumors while minimizing Laser energy absorption in surrounding healthy tissue^26^. Furthermore, complex systems of doxorubicin physically adsorbed on GNPs coated with hyaluronic acid were previously prepared seeking non-generalized systemic toxicity and offering what they called “a pinpoint drug released systems”^27,28^. Meanwhile, the authors reported 40% doxorubicin release after 12 h with hyaluronidase action, jumping to 80% following Laser near IR (NIR) stimulation.

Herein, we aim at building a combinatorial system comprising TZB, a HER2 targeting molecule, modified poly(DL-lactide-*co*-glycolide) (PLGA) and GNPs to combine targeted anticancer activity with photothermal potential for breast cancer therapy. The natural compound (Mag) with wide safety margin on normal cells^29^ and high cytotoxicity on cancer cells was used as a model anticancer drug. Integration of D-α-tocopherol polyethylene glycol 1000 succinate (TPGS), in a single step procedure, into the polymer matrix as a safe stabilizer in nanoprecipitation method relied on its ability to modify the matrix characteristics *viz* drug encapsulation efficiency, size and stability beside its reported antioxidant and anticancer properties with *P*-glycoprotein (*P*-gp) mediated multidrug resistance (MDR) inhibiting effect^13,30,31^. Facing the potential challenges and difficulties arising during formulation, while addressing the drug delivery problems, was the major study focus.

## Results

A single modified nanoprecipitation method was adopted to prepare NPs composed of a blended matrix made of PLGA and TPGS and *co*-incorporating Mag and GNPs. A particle size (PS) <200 nm with a sufficiently high zeta potential (*ζ*) to maintain colloidal stability were set as targets.

### TZB surface modified PLGA NPs

Table 1 shows that raising TPGS concentration from 0.03 to 0.18% w/v in the aqueous phase led to insignificant effect on PS as demonstrated by comparing F1 (110.6 ± 4.0) to F2, F3 and F4 (p˃0.05). It was only at a TPGS concentration of 0.24% w/v (F5) that PS increased significantly (p ≤ 0.05) scoring a value of 125.8 ± 5.5 nm. Addition of Mag (20%) to (F4) resulted in insignificant effect on PS and polydispersity index (PDI) (p˃0.05), while scoring an entrapment efficiency (EE)% and loading capacity (LC)% of 85.2 ± 0.9 and 11.36 ± 0.12% w/w, respectively, in F6. Following TPGS placement in the organic phase, no significant change was noted in PS, PDI and EE% (p˃0.05) as delineated by comparing F6 and F7 while ζ decreased significantly (p˂0.05) from −25.4 ± 1.2 (F6) to −17.5 ± 1.3 mV (F7), data not shown in table. Varying the organic to aqueous volume ratio from 1:2 (F7) to 1:1 (F8) or decreasing theoretical drug loading from 20% (F8) to 4% w/w (F9) did not significantly affect (p > 0.05) neither PS nor EE. Conversely, further rise in loading amount to a theoretical loading of 40% w/w (F10) was accompanied with significant increase (P ≤ 0.5) in PS with decline in EE%.

**Table 1:** Characteristics of PLGA NPs and Mag-PLGA NPs prepared by nanoprecipitation with and without surface modification. PLGA conc: 1.25% w/v in acetone. Results are mean of three determinations ±SD.

Code	TPGS conc (% w/v)	TPGS placement	Org:aq^a^	Mag (% w/w)^b^	PS (nm)	PDI	EE (% w/w)	LC (% w/w)	TZB CE (% w/w)
**F1**	0.03	Aqueous	1:2	**—**	110.6 ± 4.0	0.11 ± 0.003	**—**	**—**	**—**
**F2**	0.06			**—**	109.3 ± 3.6	0.11 ± 0.005	**—**	**—**	**—**
**F3**	0.12			**—**	114.9 ± 2.3	0.12 ± 0.011	**—**	**—**	**—**
**F4**	0.18			**—**	118.8 ± 3.7	0.10 ± 0.011	**—**	**—**	**—**
**F5**	0.24			**—**	125.8 ± 5.5	0.11 ± 0.021	**—**	**—**	**—**
**F6**	0.18			20	115.1 ± 2.2	0.101 ± 0.008	85.2 ± 0.9	11.4 ± 0.1	**—**
**F7**		Organic			119.5 ± 2.8	0.104 ± 0.046	80.4 ± 4.3	10.7 ± 0.6	**—**
**F8**			1:1		118.5 ± 4.8	0.104 ± 0.012	85.7 ± 1.3	11.4 ± 0.2	**—**
**F9**				4	113.0 ± 5.0	0.089 ± 0.009	95.9 ± 8.8	3.0 ± 0.3	**—**
**F10**				40	185.7 ± 4.6	0.093 ± 0.097	45.1 ± 5.0	10.6 ± 1.2	**—**
**F11**				20	145.1 ± 1.8	0.108 ± 0.020	85.1 ± 1.3	11.4 ± 0.2	61.0 ± 5.2

^a^organic to aqueous phase volume ratio, ^b^Mag percentage based on polymer weight. -: not applicable.

**Figure 1:**
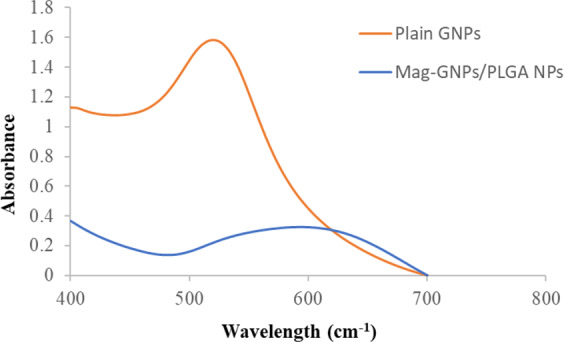
UV-Vis spectra of plain GNPs and Mag-GNPs/PLGA NPs.

GNPs were successfully prepared adopting the Turkevish method, Fig S2 (Supplementary Information)^32^. Table 2 shows the effect of GNPs incorporation in Mag-PLGA NPs prepared with TPGS. Placing GNPs dispersion in the aqueous phase failed to encapsulate GNPs in PLGA matrix as delineated by the rapid formation of a black precipitate following preparation. Conversely, GNPs addition to the organic phase, formulae P2 and P3, resulted in NPs with small uniform size as evidenced by their low PDI. Formulae P1 to P3 showed high EE ~87% w/w, comparable to that obtained *prior* to GNPs addition and exhibited non-significantly different ζ (p > 0.05) of more than −23 mV. P3, containing higher amount of GNPs, showed black precipitate upon standing, and was excluded from further study electing P2 for TZB anchoring. GNPs exhibited a surface plasmonic resonance (SPR) band at ~520 nm while Mag-GNPs/PLGA NPs (Formula P2) exhibited a shift of ~80 nm in λ_max_ accompanied with SPR broadening starting from 500 to 670 nm (UV-vis spectrum, Fig. 1). Worth to mention that neither Mag, nor plain NPs/Mag loaded NPs exhibited any absorption in the scanned range (400–800 nm).

**Table 2:** Characteristics of Mag-GNPs/PLGA NPs with and without surface modification.

Code	GNPs (µL)	GNPs conc (mM)^a^	GNPs placement	PS (nm)	PDI	*ζ* (mV)	Mag EE (% w/w)	TZB CE (%w/w)
**P1**	250	1	AqueousPhase	Black ppt	ND	−23.3 ± 1.25	88.7 ± 0.9	—
**P2**	25	10	Organic phase	127.4 ± 0.2	0.078 ± 0.06	−23.8 ± 2.8	87.2 ± 2.4	—
**P3**	50			129.3 ± 1.9	0.122 ± 0.04	−24.7 ± 0.9	88.7 ± 0.7	—
**P4**	25			136.1 ± 1.3	0.109 ± 0.009	−8.2 ± 1.0	81.4 ± 1.8	66.8 ± 3.7

Formulae were prepared at theoretical Mag loading of 5 mg, PLGA 1.25% w/v in acetone and TPGS 30% w/w of the polymer and the ratio of organic to aqueous phase was 1:1. ^a^ initial conc of added GNPs, ND: not determined due to the black ppt noted in the dispersion, -: not applicable.

Tables 1,2 show the characteristics of TZB conjugated NPs (surface modified PLGA NPs), F11 and P4 prepared without and with GNPs, respectively. Significant PS increases were noted compared to their respective unmodified counterparts F8 and P2. TZB was successfully conjugated to the surface of Mag-PLGA and Mag-GNPs/PLGA NPs with respective calculated conjugation efficiency (CE) of 61.0 ± 5.2% and 66.8 ± 3.7%w/w, respectively.

### Transmission electron microscope (TEM) examination and energy dispersion X-ray (EDX) analysis

**Figure 2:**
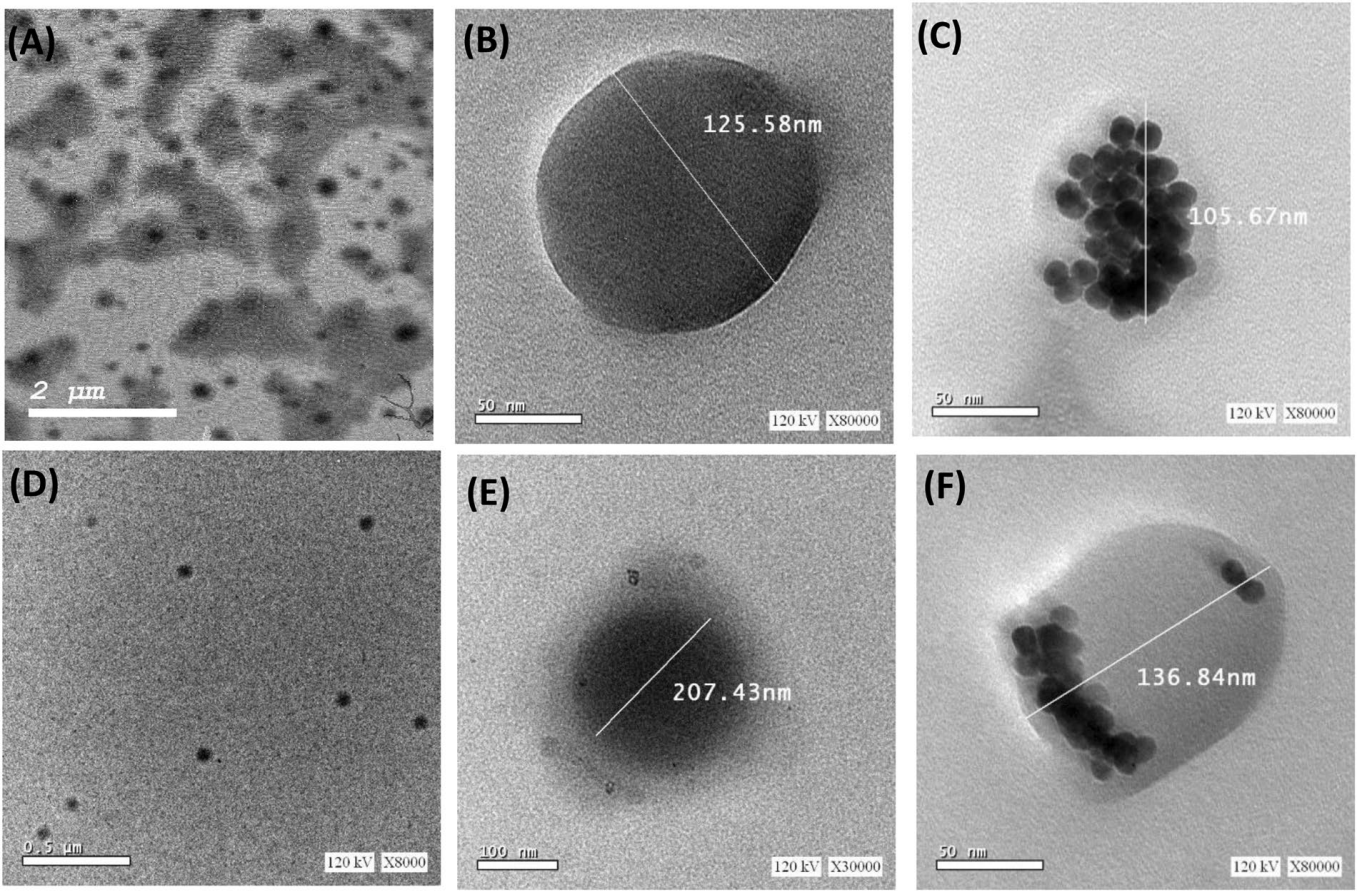
TEM micrographs of (**A,B**): Mag-PLGA NPs (formula F8) at different magnifications, (**C**): Mag-GNPs/PLGA NPs (formula P2), (**D,E**): TZB conjugated Mag-PLGA NPs (formula F11) at different magnifications and (**F**): TZB conjugated Mag-GNPs/PLGA NPs (formula P4). Note the difference in scale bars.

TEM micrographs, Fig. 2A–C, reveal that Mag-PLGA NPs and Mag-GNPs/PLGA NPs were spherical, uniform in shape with size ~100 nm. GNPs were seen as electron rich darker spheres inside PLGA matrix of Mag-GNPs/PLGA NPs. TZB conjugated NPs, showed a thicker “corona” surrounding the surface of antibody coated NPs (Fig. 2D–F). Table S1 and Fig. S3 (supplementary Information) reveal that O and Au were the main elements present within TEM-EDX inspection field of Mag-GNPs/PLGA NPs taking into consideration that %C was omitted as the copper grid used in EDX analysis was carbon coated.

### In vitro drug release and NPs stability in serum

Very low Mag burst release amounting of 6.52 ± 1.94 and 1.76 ± 0.37% was noted with Mag–PLGA (F8) and Mag-GNPs/PLGA (P2) NPs formulae, respectively, during the first two hours of the release experiment, Fig. S4. In contrast to pure Mag powder, which showed 97% release in 48 h, PLGA NPs successfully sustained the drug release with 54.7 ± 1.60 and 50.24 ± 2.41 being released from Mag–PLGA NPs (F8) and Mag-GNPs/PLGA NPs (P2), respectively, during 48 h. The aggregation and dimensional growth produced by deposition of serum proteins on particles surface of selected formula P4 were assessed by the change in NPs dimension monitored by DLS. Mag-GNPs/PLGA-TZB NPs did not show any significant change in PS (Table S2) following storage in serum for 4 h.

### Fourier transform-infrared (FTIR) and Proton nuclear magnetic resonance (^1^HNMR)

PLGA FTIR spectrum, Fig. S5A, shows C=O and OH stretching at 1758 and ~3500 cm^−1^, respectively, while TPGS spectrum shows a carbonyl band at 1738 cm^−1^. Mag-PLGA NPs spectrum reveals the disappearance of Mag fingerprint along with reduction of Mag OH stretching peak ~3500 cm^−1^. Mag-GNPs/PLGA NPs spectrum shows a broad peak at ~ 3400–3500 cm^−1^ of higher intensity compared to Mag-PLGA NPs referred to citrate OH group (Fig. S5C). About 65 and 73 cm^−1^ shifts in PLGA carboxylic acid OH stretching peak from 3488 to 3423 and 3415 cm^−1^ were obvious in TZB surface modified NPs (Mag PLGA-TZB NPs and Mag-GNPs/PLGA-TZB NPs, respectively) spectra compared to their unconjugated ones, (Fig. S5B,C). Furthermore, TZB finger print region in TZB modified NPs is evident.

Figure S6A shows typical TPGS ^1^H-NMR spectrum where –CH_2_ protons peak is evident at 3.65 ppm. Fig. S6B, distinctly shows PLGA proton peaks at 5.10, 4.82 and 1.655 ppm, corresponding to –CH, –CH_2_ and –CH_3_ protons of PLGA segments along with TPGS proton peaks at 3.65ppm. TZB ^1^H-NMR spectrum (Fig. S6C) reveals the presence of a peak at 3.5ppm corresponding to -NH_2_ protons together with characteristic peaks at δ values of 7.3, 7.27, 7.24 ppm corresponding to TZB aromatic group. Mag PLGA-TZB NPs ^1^H-NMR, Fig. S6D, reveals similar peaks at (δ = 7.311, 7.277, 7.242 ppm).

### MTT assay

**Figure 3:**
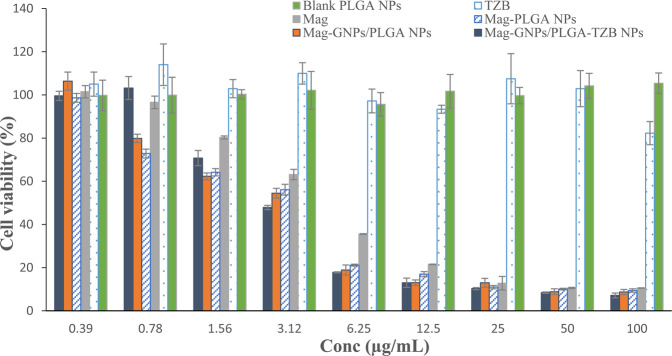
MCF-7 breast cancer cell viability measured by MTT cytotoxicity assay after exposure to increasing concentration of Mag solution, TZB solution, blank NPs, Mag-PLGA NPs, Mag-GNPs/PLGA NPs and Mag-GNPs/PLGA-TZB NPs. *Blank NPs were used at the same solid content concentration of their equivalent medicated counterpart. The results shown were the mean of three independent experiments, each performed in triplicate (n = 9).

Figure 3 reveals that no significant decrease in cell viability occurred when MCF-7 cells were treated with both blank PLGA NPs (at all tested concentrations) and TZB solution (up to 50 µg/mL) during the 24 h dose-dependent (0–100 µg/mL) study. Conversely, gradual decreases in viability from 100% to 9.41 ± 0.8 and 8.73 ± 1.19% were found with Mag-PLGA NPs and Mag-GNPs/PLGA NPs, respectively. The viability of cells exposed to both NPs formulae were insignificantly different among each other (p > 0.05) while significantly (p ≤ 0.05) lower than that of pure Mag solution at the same concentration. The calculated IC_50_ for Mag was found to be 2.92 ± 0.32 µg/mL and this value significantly decreased to 1.81 ± 0.02, 2.22 ± 0.44 and 1.76 ± 0.10 µg/mL following its incorporation in PLGA NPs, GNPs/PLGA NPs and GNPs/PLGA-TZB NPs, respectively, Table 3.

**Table 3:** Calculated Mag IC_50_ from various formulations in MCF-7 cells. Data were calculated based on the mean of 3 experiments each performed in triplicate.

Photo- irradiation	Formula	IC_50_ (µg/mL)
None	Mag solution	2.92 ± 0.32
	Mag/PLGA NPs	1.81 ± 0.02
	Mag-GNPs/PLGA NPs	2.22 ± 0.44
	Mag-GNPs/PLGA-TZB NPs	1.76 ± 0.10
Cells were photo-irradiated with LED at a wavelength of 650 nm for 45 min	Mag-GNPs/PLGA NPs	1.34 ± 0.01
	Mag-GNPs/PLGA-TZB NPs	1.10 ± 0.07

### Photothermal (PT) effect

**Figure 4:**
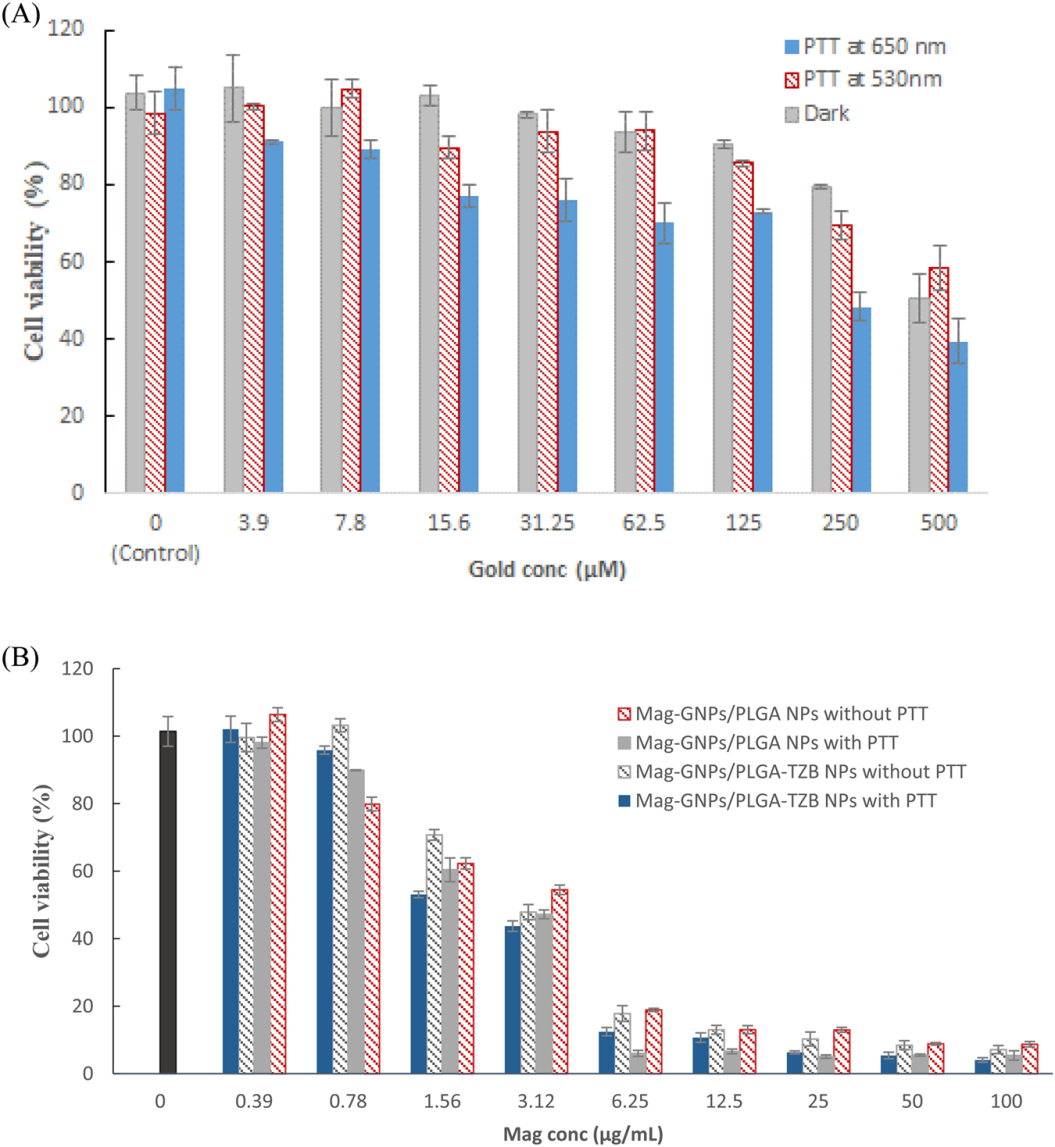
Effect of photo-irradiation using LED at 650 nm on the viability of MCF-7 breast cancer cell viability after exposure to increasing concentration of (**A**) GNPs and (**B**) Mag-GNPs/PLGA NPs. The results shown were the mean of three independent experiments, each performed in triplicate (n = 9).

A preliminary study showed that MCF-7 cells treatment with GNPs (3.9 μM gold) for 24 h resulted in viabilities of 105.75 ± 8.84 and 91.55 ± 0.77% in dark and following photo-irradiation at 650 nm, respectively (Fig. 4A). Increasing gold concentration to 250 µM considerably decreased viability, which reached 79.25 ± 0.49% and 48.37 ± 3.80%, respectively at both conditions. Worthy to mention here that testing LED photo-irradiation at two wavelengths (530 and 650 nm) revealed that, only, 650 nm resulted in significant decrease in MCF-7 cells viability relative to the experiments in dark, Fig. 4A, and the decrease was accentuated at gold concentrations ≥62.5 μM. When untreated MCF-7 were exposed to monochromatic LED at 650 nm (red light) for 45 min, no decrease in cell viability was observed compared to the control cells (unexposed to light). Meanwhile, Mag-GNPs/PLGA NPs showed decrease in cell viability from 98.06.58 ± 1.64 to 5.40 ± 1.59% with increasing NPs concentration equivalent to Mag concentration from 0.39 to 100 µg/mL after red LED irradiation. The observed viabilities were significantly lower following photo-irradiation with the NIR light compared to non-photo-irradiated cells treated with similar NPs concentrations (Fig. 4B). The calculated IC_50_ of Mag-GNPs/PLGA NPs and Mag-GNPs/PLGA-TZB NPs showed significant decreases (*P* < 0.05) from 2.22 and 1.75 (no PT) to 1.34 ± 0.01 and 1.10 ± 0.07 µg/mL (with PT) respectively, Table 3.

### Cellular uptake and internalization of TZB surface modified and unmodified Nile red-GNPs/PLGA NPs

**Figure 5:**
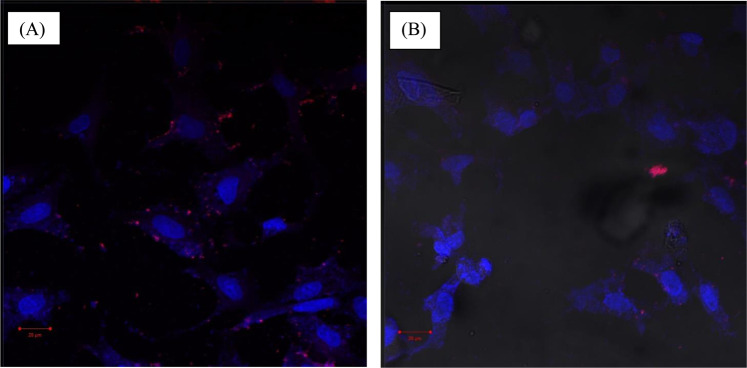
CLSM images of MCF-7 cells treated for 15 min with (**A**) TZB modified Nile red-PLGA/GNPs and (**B**) non-surface modified Nile red-PLGA/GNPs. The nuclei were stained with DAPI and the NPs were labeled with Nile red. The scale bar shown represent 20 µm.

Samples of confocal scanning laser microscopy (CLSM) images show uptake of GNPs/PLGA NPs inside MCF-7 cells and their localization in the cytoplasm (Fig. 5). A time dependent increase in fluorescence intensity until 2 h post NPs incubation with cells was observed. After 2 h incubation, Nile red labeled NPs were well distributed around blue stained nuclei. CLSM images, Fig. 5, show that TZB modified NPs (GNPs/PLGA-TZB) underwent a relatively faster uptake (within 15 min of incubation) compared to the non-targeted analog. CLSM images showing serial z-sections of the cells, each ~0.1 µm in thickness (Figs. S7 & S8), demonstrated high fluorescence activity of both non modified and TZB modified Nile red-GNPs/PLGA NPs in sections between 3.71 and 8.36 µm from cell surface.

## Discussion

A combinatorial nanocarrier system with a target size smaller than 200 nm suitable for passive cancer targeting utilizing enhanced permeability and retention was first constructed^33^.To endue active targeting specificity to HER2 overexpressed breast cancer cells, TZB was used to decorate carrier surface. A systematic multistep approach was used to construct the proposed Mag carrier and conquer the formulation difficulties pertaining to the development of a multicomponent system.

Designing a matrix comprising TPGS was targeted for providing, not only a stabilizing effect but also, to endue stealth properties to NPs besides its known anticancer properties^34^. To find the appropriate concentration for NPs stabilization, TPGS was first placed in the aqueous phase, and the highest concentration of TPGS providing the smallest size was selected; relying on the reported TPGS role in inhibiting of *P-gp* mediated MDR. Accordingly, F4 was selected for drug loading study, Table 1. Based on its amphiphilic nature^31^, an attempt to place TPGS in the organic phase was done to allow its incorporation in the NPs matrix providing a lipid polymer modified core. Interestingly, size and drug EE were not significantly affected compared to its placement in the aqueous phase, while the decrease in ζ between F6 and F7 confirmed a better TPGS coating onto the PLGA NPs surface providing a possible stealth effect on Mag NPs^13^. The appearance of a new band at 1759cm^−1^ in PLGA/TPGS blank FTIR spectrum compared to PLGA (Fig. S5A), with the change in position of PLGA hydroxyl groups at 3500–3650 cm^−1^, confirmed PLGA interaction with TPGS and formation of a new PLGA/TPGS matrix. A further confirmation could be withdrawn from ^1^HNMR, Fig. S6, showing presence of TPGS -CH_2_ protons peak.

A 20%w/w theoretical drug loading was selected as higher drug concentration probably resulted in a more viscous dispersed phase producing larger particles with a lower EE, Table 1. Due to its hydrophobic nature, Mag was highly encapsulated within the composite matrix providing high LC. Formula F8, prepared at Mag (5 mg equivalent to 20%w/w drug loading) and showing small PS (118.5 ± 4.8 nm), high EE% (85.7 ± 1.3%) with the highest LC% (11.4 ± 0.2%), was selected for preparation of immuno-NPs. TEM, Fig. 2A,B, shows that the size of the obtained NPs was in accordance with dynamic light scattering (DLS) data. Mag encapsulation was evidenced by the disappearance of its fingerprint along with reduction in OH stretching peak ~ 3500 cm^−1^ in FTIR spectrum, Fig. S5. The relatively fast Mag release (39.64 ± 2.46% w/w) from PLGA/TPGS NPs during the first day, Fig. S4, might be attributed to the presence of the amphiphilic TPGS^13^, causing rapid absorption of water molecules into the polymeric matrix, hence promoting drug diffusion through the integrated lipid-polymer matrix. This was an another advantage of PLGA/TPGS NPs over traditional PLGA NPs, which were found to slowly release the drug below therapeutic needs in some cases^35^.

**Scheme 1:**
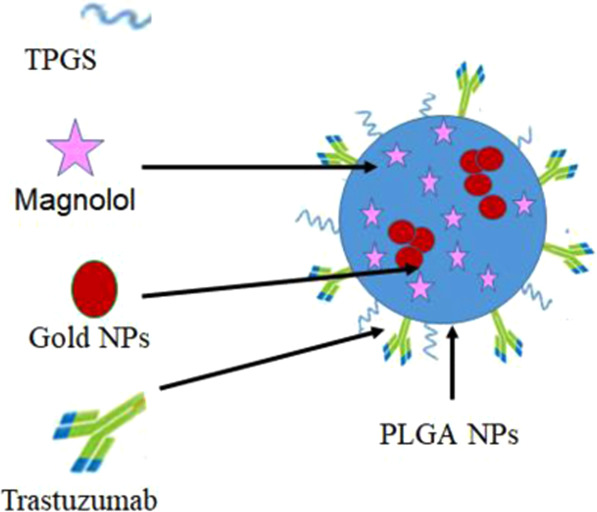
Proposed diagram for the developed Mag-GNPs/PLGA-TZB NPs.

Two methods were attempted for *co*-encapsulating GNPs within the modified PLGA-TPGS matrix, Table 2. While, its placement in the aqueous phase compromised the system stability where black precipitate was noted, placing GNPs in the organic phase was only successful following the use of concentrated dispersions. A small volume (25–50 µL) could only be incorporated in organic phase before precipitation and/or improper dissolution of PLGA could be noticed. The significant decrease in ζ recording negative values of ~23–24 mV (formulae P2-P3) following incorporation of GNPs denoted its adsorption on PLGA NPs surface. Furthermore, comparing UV absorption spectra of GNPs before and after incorporation within NPs, the shift noted in GNPs λ_max_ accompanied with SPR broadening starting from 500 to 670 nm indicated clustering of GNPs into polymeric nanoconstructs^36^. A schematic illustration of the developed smart Mag-GNPs/PLGA-TZB NPs is represented in Scheme 1.

TEM micrographs, Fig. 2C,F, show GNPs as electron rich darker spheres inside PLGA matrix of Mag-GNPs/PLGA NPs and high percent of Au in TEM-EDX, Fig. S3 & Table S1, confirmed the encapsulation of GNPs inside PLGA. Obviously *co*-encapsulating GNPs with Mag in PLGA NPs did not greatly affect the drug release where a sustained release profile could be achieved in both presence and absence of GNPs, Fig. S4.

TZB was highly conjugated to NPs formulae F11 and P4 as delineated by the calculated amount of TZB in NPs in addition to the significant increase in PS with decrease in ζ noted compared to their counterparts free from TZB, F8 (Table 1) and P2 (Table 2). A significant decrease (*P* < 0.05) in ζ was also noted with P4 compared to P2 with respective values of −8.2 ± 1.0 and −23.8 ± 1.3 mV. Evidence could also be provided from the FTIR spectrum showing TZB fingerprint, in addition to the carboxylic OH shift (Fig. S5B,C) and ^1^H NMR spectrum showing TZB aromatic protons at (7.311, 7.277 and 7.242 ppm, Fig. S6C,D). These NPs are expected to be stable in biological medium following intravenous administration as demonstrated by the absence of aggregates or noticeable PS change following their incubation in serum for 4 h, Table S2. This could be referred to the high negative ζ, beside the steric stabilization probably provided by TPGS and TZB surface modification.

MTT assay, Fig. 3, confirms the biocompatibility and tolerability of blank PLGA NPs prepared with TPGS on MCF-7 cell line at all tested concentrations. Similarly, TZB was found to be safe up to a concentration of 50 µg/mL. TZB had been reported to prevent cell proliferation rather than inducing cell death and, in accordance with our observation, an IC_50_ of >200 µg/mL for TZB was previously reported^37^. On the other hand, Mag IC_50_ was found to decrease from 2.92 ± 0.32 for Mag solution to 1.81 ± 0.02 µg/mL following its incorporation into NPs, Table 3. Furthermore, conjugating TZB to drug loaded NPs did not considerably affect the cytotoxicity where an IC_50_ value of 1.755 ± 0.10 µg/mL was recorded with Mag-GNPs/PLGA-TZB. This was non-significantly (P˃0.05) different from non-surface modified NPs. However, a relatively faster uptake of modified NPs was noted in CLSM images compared to the unmodified ones, Fig. 5. A relatively high concentration of fluorescent NPs could be internalized after 15 min, in case of TZB surface modified NPs. Conversely, the red fluorescence was seen within the cells at high intensity only around 60 min in case of unmodified NPs. In the present study, due to their small size (˂200 nm), both TZB surface modified and unmodified Mag GNPs/PLGA NPs, were allowed to enter the cell through the more fluid cell membrane, a known characteristic of cancer cells. In case of TZB modified NPs, an active interaction between the mAb present on the nanocarrier surface with HER2 expression on MCF-7 cell surface might be expected. The intensity of red fluorescence increased with incubation time, suggesting increased uptake of NPs by lysosomes followed by possible release of Nile red into the cytoplasmic compartment. These results indicate that TZB targeted NPs can undergo receptor-mediated rapid internalization in MCF-7 cells, in spite of the low expression of HER2. Due to the absence of the mAb from the NPs surface in the non-targeted formulation, NPs cannot undergo receptor mediated internalization, and the cellular uptake occurred through slower processes like lipid rafts, membrane fusion, pores, as well as through caveolae. Thus, CLSM images showed difference in the speed of uptake between TZB modified and unmodified NPs in spite of the small non-significant difference in cell cytotoxicity. The recorded CLSM images showing the z-sections denoted that NPs were transported from the apical surface of the cell membrane towards the basolateral membrane confirming that NPs were indeed inside the cytoplasm and not simply adsorbed to the outer surface (Fig. S7&S8). Worthy to highlight that the use of MCF-7 cells with low to moderate expression of HER2 stood behind the small noted difference between the cellular uptake of two NPs formulae (TZB surface modified and unmodified)^2,12,30^. We can expect a higher increase in drug cytotoxicity with a greater decrease in drug IC_50_ and a higher effect for TZB in targeting efficiency while using breast cancer cells with high level of HER2 expression^1^.

In accordance with previous reports, GNPs exhibited no intrinsic cytotoxic or anti-proliferative effects on cells as delineated by MTT assay, Fig. 3^38^. Following PT irradiation of GNPs, it was only at 650 nm that a significant decrease in MCF-7 cells viability compared to the experiments in dark was noted. The clustering of GNPs in PLGA NPs or in MCF-7 cells provided a significant shift of plasmonic resonance to NIR range that may make 650 nm application more effective with spherical GNPs. Previous works suggested that GNPs had a tendency for self-assembly in clusters *in vitro* as well as *in vivo*^39^. It is also to be noted that it was at high GNPs concentration that the effect of irradiation was prominent. Worthy to mention that used light source was safe at the tested power, 200 mW, on untreated MCF-7 cells. However, the 1.6 fold significant decrease in IC_50_ following photo-irradiation of cells treated with Mag-GNPs/PLGA NPs and Mag-GNPs/PLGA-TZB NPs confirmed the responsiveness of the developed system to a NIR treatment and its ability to boost the drug cytotoxic effect. The decrease in cell viability can also be tuned by adjusting the amount of GNPs as delineated from Fig. 4A. Higher gold concentration resulted in a greater decrease in cell viability when the cells were exposed to LED 650 nm.

## Conclusions

Magnolol was highly loaded in the composite nanoconstruct matrix with an encapsulation efficiency exceeding 81%. Meanwhile, TZB was highly conjugated to the NPs with an efficiency reaching ~66.8% in presence of *co*-encapsulated magnolol and GNPs. The clustered GNPs, imparted a photothermal near IR response to the developed nanocarrier, boosting the drug cytotoxicity and augmenting the specificity to cancer cells. Conclusively, Mag-GNPs/PLGA-TZB NPs could be considered a breast cancer smart multifunctional therapy showing notable combined targeted anticancer activity with a photothermal potential holding promises for less unnecessary side effects.

## Materials and Methods

### Materials

1-ethyl-3-(3-dimethylaminopropyl) carbodiimide hydrochloride (EDC): Thermo Fisher Scientific, UK. 3-(4,5-dimethylthiazol-2-yl)−2,5-diphenyl-tetrazolium bromide (MTT), penicillin G sodium, streptomycin sulfate, diamidino-2-phenylindole dihydrochloride (DAPI), dimethyl sulfoxide (DMSO), gold chloride (HAuCl4) and N-hydroxysuccinimide (NHS) 98%, Nile red: Sigma, UK. Poly(DL-lactide-*co*-glycolide) (PLGA) 50/50, grade 5002 A, viscosity 0.2 dl/g, acid terminated: kindly provided by Corbion Purac Biomaterials, The Netherlands. Bicinchonininc acid (BCA) protein assay reagent kit: Pierce, USA. Citifluor PVA plus antifade: Citifluor Ltd, UK. D-α-tocopherol (vitamin E) polyethylene glycol 1000 succinate (TPGS): kindly provided by Isochem, France. Rosewell Park Memorial Institute medium (RPMI-1640), phenol red free RPMI and fetal bovine serum (FBS): Gibco, UK. Herceptin (Trastuzumab (TZB)): monoclonal antibody (mAb) obtained from F. Hoffmann-La Roche Ltd, Switzerland. Magnolol (Mag): Xian Lyphar Biotech Co., Ltd, China. Paraformaldehyde 16% aqueous solution: Alfa Aesar, UK. Sodium dodecyl sulphate (SDS): BDH Laboratories Supplies, UK. MCF-7 cell line with moderate expression of HER2 receptors were obtained from Vaccera, Egypt.

### Synthesis of citrate capped-gold NPs (GNPs)

Colloidal GNPs were synthesized according to the Turkevich method using gold chloride (HAuCl4) and sodium citrate as precursor and reducing agent, respectively^32^. Briefly, HAuCl4 solution (100 mL) was boiled in a conical flask followed by addition of 1% w/v sodium citrate aqueous solution (10 mL). Heating under reflux with stirring continued till formation of a red color^40^.

### Optimization of PLGA NPs co-encapsulating Mag and GNPs

PLGA NPs were prepared by a modified nanoprecipitation method^33^. Briefly, PLGA was dissolved in acetone (2 mL), which was then dripped into the aqueous phase maintained under magnetic stirring at 250 rpm. TPGS placement was attempted in both aqueous and organic phase, Table 1. The organic solvent was removed by stirring at 100 rpm at room temperature. NPs were purified using two cycles of centrifugation (Centurion K241, Germany) at 30,000 rpm for 30 min at 4 °C followed by washing with deionized water. Pellets were dispersed in 2 mL water and the dispersion was frozen at −20 °C then freeze dried (Christ alpha 1–2 LD plus, Germany). For drug loading, accurately weighed Mag, PLGA and TPGS were added to the organic phase and the procedure was completed as before. To achieve the highest drug loading, while maintaining optimum size and uniformity, formulation parameters were varied (Table 1).

To *co*-encapsulate GNPs in Mag-PLGA NPs, attempts to disperse GNPs either in aqueous or organic phase were performed. In the former method, acetone containing Mag, PLGA and TPGS was dripped into the aqueous phase containing colloidal GNPs (1 mM) placed under magnetic stirring at 250 rpm and the procedure was completed as before. In the second method, different volumes of GNPs dispersion (10 mM), obtained by concentrating 1 mM gold dispersion by centrifugation at 4000 rpm and room temperature using *Nanoseps* (MwCO 10 KDa, Pall, USA), were mixed with the organic phase which was then dripped on the aqueous phase^41^. Table 2 shows the composition of the developed formulae.

### Preparation of immuno-PLGA NPs using covalent conjugation

Mag-PLGA NPs and Mag-GNPs/PLGA NPs were surface modified with the mAb, TZB, implementing a covalent binding method^42^. Briefly, freeze dried NPs were dispersed in 5 mL water (pH adjusted to 5) at a concentration of 5 mg/mL, followed by addition of 20 mg of each of EDC and NHS and the dispersion was stirred at 150 rpm for 30 min. Excess NHS and EDC were removed by dialysis against 100 mL deionized water using dialysis membrane (MwCO 14 KDa,Thermo Fisher Scientific, UK) overnight with frequent replacement of water. NPs were concentrated using *vivaspin* columns (MwCO 10 KDa, Sartorius Stedim Lab Ltd, UK) for 30 min at 4 °C, resuspended in 5 mL phosphate buffer solution (PBS), pH 7.4. Then 250 µL of TZB (10 mg/mL) solution in PBS pH 7.4 were added to the activated pellet while stirring at room temperature at 50 rpm for 4 h. TZB bound NPs were separated from free TZB by centrifugation. The NPs were washed with deionized water, re-centrifuged, then re-dispersed in 2 mL of PBS, pH 7.4.

### PS, PDI and ζ determination

PS, PDI and ζ measurements were carried out on the freshly prepared NPs dispersions using DLS for PS and PDI and laser Doppler velocimetry for ζ using a Zetasizer (Nano ZS, Malvern, UK).

### Spectral analysis

Scanning of GNPs and Mag-GNPs/PLGA NPs using UV/Vis spectrophotometer (UV-1601PC, Shimadzu, Japan) in the range of 400 to 800 nm was conducted and their SPR was determined. Mag solution in 1:1 (water: ethanol) mixture as well as Mag-PLGA NPs and plain PLGA NPs were also treated similarly.

### TEM examination

TEM images were recorded on TEM equipped with EDX (JEM 1400 plus, JEOL, Japan) at 120 Hz.

### Determination of Mag EE and LC

Accurately weighed (10 mg) of freeze dried NPs were suspended in 4 mL of 1%w/v NaOH solution containing 0.5%w/v SDS^43^ and the mixture was left for 24 h at 37 °C on a magnetic stirrer. Mag concentration was determined using reversed phase RP HPLC-UV method (Jasco, 2080 plus, Japan) using a platinum C18 column at 25 °C. Samples were eluted with an isocratic system composed of acetonitrile: 0.1% w/v phosphoric acid (65:35) at flow rate of 1 mL/min with UV detection at λ_max_ = 292 nm^44^.

1 $${\rm{EE}}\, \% =({\rm{Amount}}\,{\rm{of}}\,{\rm{Mag}}\,{\rm{in}}\,{\rm{NPs}}/{\rm{Initial}}\,{\rm{amount}}\,{\rm{of}}\,{\rm{Mag}})\times 100$$

2 $${\rm{LC}}=({\rm{Amount}}\,{\rm{of}}\,{\rm{Mag}}\,{\rm{in}}\,{\rm{NPs}}/{\rm{NPs}}\,{\rm{weight}})\times 100$$

### Determination of *in vitro* drug release

Accurately weighed Mag-NPs equivalent to 2 mg Mag, dispersed in 2 mL PBS (pH 7.4), were placed inside a dialysis sac (MWCO 14 KDa) which was tied at both ends and was immersed in a beaker containing 50 mL of PBS, pH 7.4, containing 0.5% w/v SDS to ensure sink conditions. The beaker was placed in shaker water bath (Labsol, India) at 80 strokes/min at 37 °C. One mL of dissolution medium was withdrawn at predetermined time intervals and was replaced with fresh medium. The amount of released drug was determined using the previously explained HPLC method.

### FTIR analysis

FTIR spectra were obtained using a spectrometer (Thermonicolet Nexus, USA). Freeze dried samples were pressed into potassium bromide (KBr) pellets and recorded at frequencies from 4000 to 200 cm^−1^ with resolution of 4 cm^−1^.

### Determination of TZB conjugation efficiency (CE)

The CE of TZB on NPs surface was determined adopting an indirect quantification method using a micro BCA protein assay kit at 562 nm. TZB amount was determined by measuring protein concentration in supernatant obtained following ultracentrifugation of NPs dispersion in *Nanoseps* 100 KDa at 6,000 rpm for 1 h at 4 °C.

3 $${\rm{CE}}( \% )=({\rm{Initial}}\,{\rm{TZB}}\,{\rm{amount}}-{\rm{amount}}\,{\rm{in}}\,{\rm{supernatant}}/{\rm{Initial}}\,{\rm{TZB}}\,{\rm{amount}}\,)\times 100$$

### ^1^H-NMR analysis

Freeze dried NPs sample was dissolved in 0.2 mL DMSO and vortexed for 48 h. Scan was done using 3 mL NMR tube, frequency 400 MHZ, pulse width 12 W and scan number 16 using a spectrometer (Bruker HD III NMR spectrometer, Switzerland).

### NPs serum stability study

Accurately weighed freeze dried Mag-GNPs/PLGA NPs amount (3 mg) was suspended in 50% v/v FBS aqueous solution (1 mL), followed by incubation at 37 °C. PS was determined at different time intervals (0, 1, 2 and 4 h) using DLS.

### Cytotoxicity evaluation

Cytotoxicity of selected Mag-PLGA NPs, Mag-GNPs/PLGA NPs and Mag-GNPs/PLGA-TZB NPs along with plain PLGA NPs, free drug solution and TZB solution were evaluated on breast cancer cells (MCF-7), using MTT assay^2,12,30^. Serial concentrations of NPs and TZB were directly prepared in serum free RPMI-1640 while a stock solution of Mag was first done in DMSO before dilution with the medium to achieve concentrations ranging from 100 to 3.9 µg/mL. Cells seeded in 96 wells plate at a concentration of 5 × 10^3^ cells/well and left to attach overnight, were treated with the assigned formulation for 24 h at 37 °C (humid CO_2_ incubator, Heraeus, Germany). Untreated cells were used as negative control. The culture supernatant was then removed and the MTT assay was conducted as previously explained elsewhere^30^. The results obtained were the mean of three independent experiments, each performed in triplicate (n = 9). Percentage viability was calculated as follows^30^.

4 $${\rm{Cell}}\,{\rm{viability}}\, \% =({\rm{A}}({\rm{test}})/{\rm{A}}({\rm{negative}}\,{\rm{control}}\,)\times 100$$

IC_50_ was calculated according to the equation for Boltzman sigmoidal concentration–response curve (Graph Pad, Prism version 5).

### PT effect

MCF-7 cells with moderate HER2 receptors expression^2^ were first treated with either plain GNPs, Mag-GNPs/PLGA NPs and Mag-GNPs/PLGA-TZB NPs, then exposed to light energy using light-emitting diode (LED) source for a specific period *prior* to MTT assay. Briefly, cells were seeded in 96-well plates containing 200 µL of phenol red free RPMI at a density of 5 × 10^3^ cells/well. Cells were permitted to adhere for 24 h till confluence in a 5% CO_2_ incubator. Serial dilutions from 500 to 3.9 µM of plain GNPs were prepared by diluting the aqueous stock solution with the culture and 200 µL of medium containing GNPs were added in each well. After 24 h of incubation, cells were washed twice with PBS to remove non-internalized GNPs, then 200 µL fresh phenol red free medium was added. Cells were then subjected to monochromatic light from LED source of 650 nm (red light) at 200 mW power for 45 min, while incubated in maintenance medium. A 24 h incubation time was given to stabilize the cells. The medium was then aspirated and replaced with fresh medium. Application of each therapeutic modality, Mag-GNPs/PLGA or Mag-GNPs/PLGA-TZB NPs, was followed by evaluation of tumor cells viability using MTT assay^30^. Results were determined from three independent experiments performed in triplicate (n = 9) and the IC_50_ was calculated.

### CLSM

Nile red labeled NPs were first prepared by the modified nanoprecipitation method, previously described using 10 μL of Nile red stock solution in acetone (1 mg/mL) instead of Mag. NPs surface was decorated with TZB as previously explained.

MCF-7 cells were seeded in 24 wells plate (5 × 10^4^ cells/200 µL/well) on 35 mm glass cover slips, incubated for 24 h and then washed with RPMI-1640 to remove non-adherent cells^5^. Cells were then incubated with Nile red loaded NPs for 15, 60 and 120 min to allow for uptake. The medium was then aspirated, and the cells were washed 3 times with PBS, fixed with 4% paraformaldehyde solution for 15 min at room temperature then washed three times with PBS. For nuclear staining, cells were covered with DAPI 300 nM prepared in PBS for 15 min in dark. For microscopical examination, the cover slips were mounted with aqueous PVA citifluor reagent mixed with AF100 antifade reagent (1:10). Slides were examined under the confocal microscope (Zeiss LSM 510 Meta) and CLSM images were taken. Red channel for Nile red: excitation: 561 nm, emission collected: 570–620 nm; blue channel for DAPI: excitation: 405 nm, emission collected: 425–475 nm*.* Z-series of optical sections were acquired at spacing steps of 0.6 µm from the surface through the vertical axis of the specimen by a computer-controlled motor drive.

### Statistical analysis

All formulations were prepared and reported in triplicate. Results are expressed as mean ± standard deviation (SD). The statistical significance of difference between groups were evaluated by one-way ANOVA and Tukey’s post hoc test with a significance level of *p* < 0.05. Graph Pad, Prism Version 5 was used for calculating IC_50_ values in cytotoxicity assay.

## Supplementary Information

Supplementary Information.
