# Supplementary material for: Near IR responsive targeted integrated lipid polymer nanoconstruct for enhanced magnolol cytotoxicity in breast cancer
Source: Sci Rep. 2020 May 29;10:8771. doi: 10.1038/s41598-020-65521-z (PMC7260181; doi:10.1038/s41598-020-65521-z)
Supplement: Supplementary file 1 — Supplementary Information. [file 41598_2020_65521_MOESM1_ESM.pdf]

Supplementary Information

Near IR responsive integrated targeted lipid polymer nanoconstruct for enhanced magnolol cytotoxicity in breast cancer

Mona Elhabak[1], Rihab Osman[2*], Mona Mohamed[3], Ola El-Borady[4], Gehanne AS Awad[2], Nahed Mortada[2]

[1]Department of Pharmaceutics and Industrial Pharmacy, Faculty of Pharmacy, Al-ahram Canadian University, Egypt.

[2]Department of Pharmaceutics and Industrial Pharmacy, Faculty of Pharmacy, Ain Shams University, P.O. Box 11566, Abbassia, Cairo, Egypt.

[3] National Institute of Laser Enhanced Science, Cairo University, Cairo, Egypt

[4] Institute of Nanoscience and Nanotechnology, Kafrelsheikh University, Kafrelsheikh, Egypt

Element	Weight (%)	Atomic (%)
O K	63.66	95.57
Au M	36.34	4.43
Totals	100	

Table S1

Percent of O and Au in Mag-GNPs/PLGA NPs determined by EDX.*O: oxygen, Au: gold, K: K* level of the X-ray emission lines for oxygen, M: M level of the X-ray emission lines for gold.

Incubation time (h)	PS (nm)
0	136.1±1.27
1	141.4±5.83
2	137.1±2.62
4	144.0±6.81

Table S2

Characteristics of Mag-GNPs/PLGA-TZB NPs (formula P4) after storage in serum (50% FBS for 4 h). Results are mean of 3 determinations (±standard deviation).

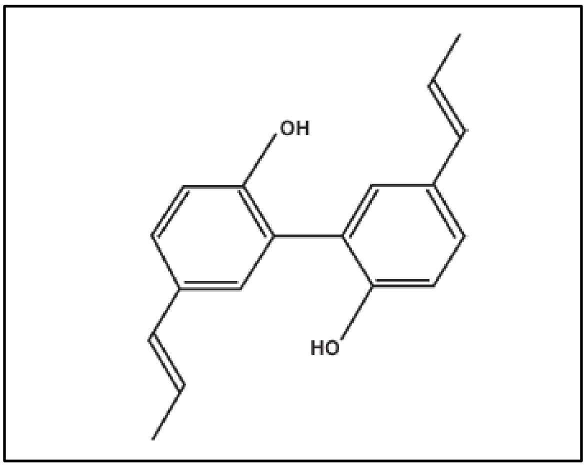

Figure S1: Chemical structure of magnolol (2-(2-hydroxy-5-prop-2-enylphenyl)-4-prop-2-enylphenol)

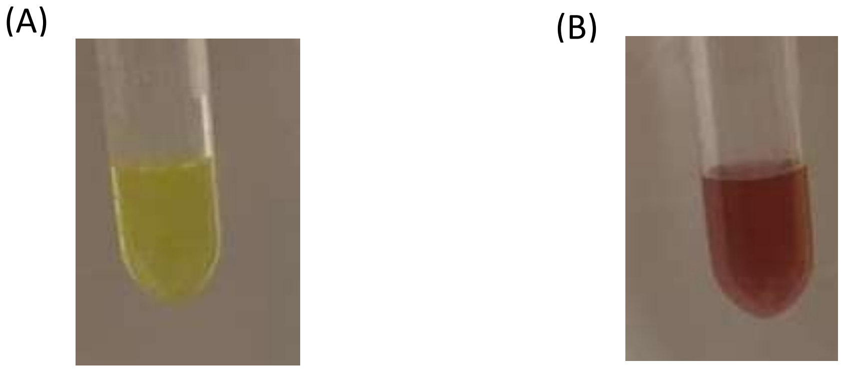

Figure S2: Photograph of gold dispersion (A) before and (B) after GNPs formation by the Turkevish method. For NPs preparation, HAuCl4 solution (100mL) was boiled in a conical flask under continuous stirring followed by addition of 1% w/v sodium citrate aqueous solution (10mL). Heating under reflux with stirring continued till formation of a red color.

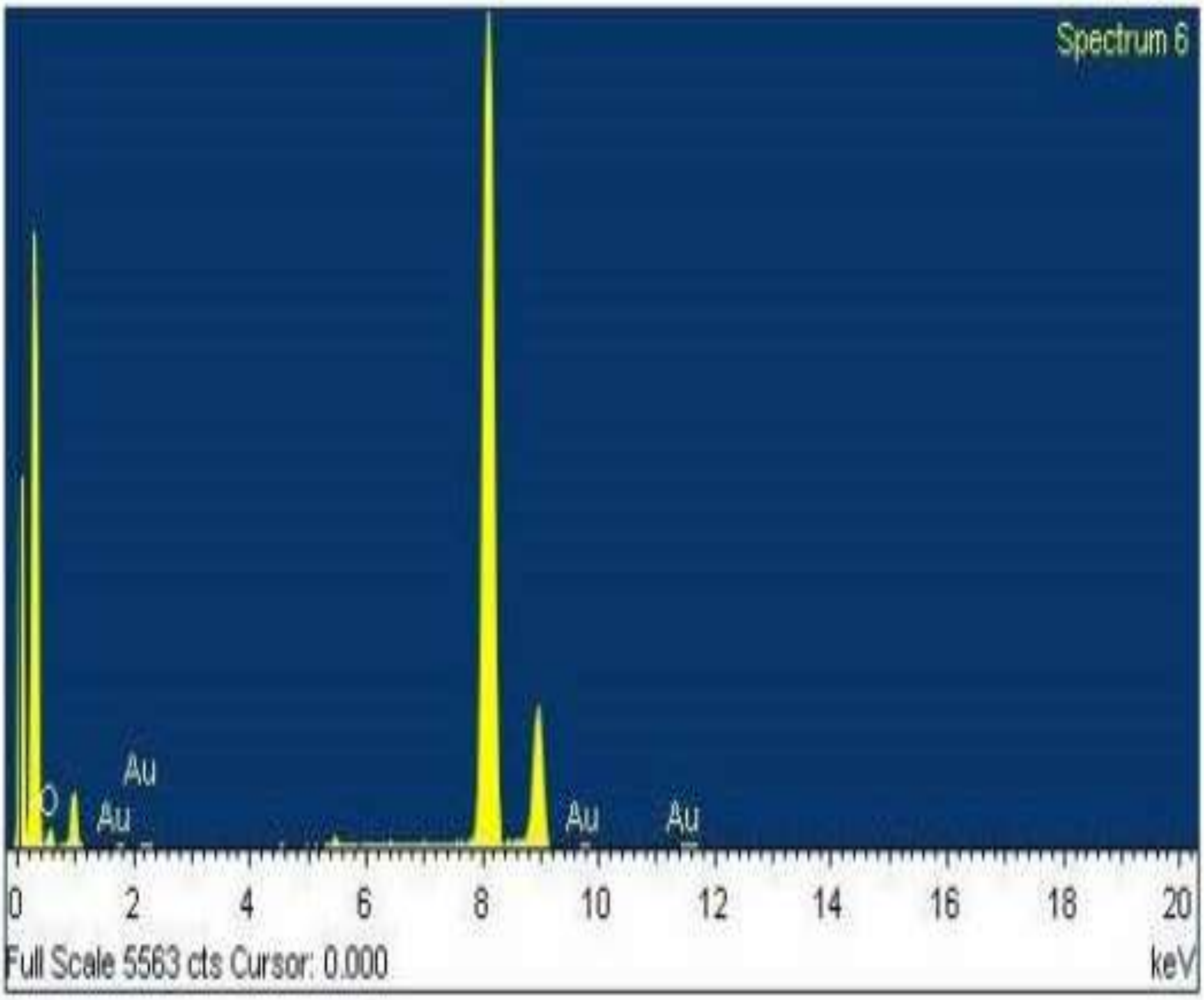

Figure S3 EDX of Mag-GNPs/PLGA NPs.

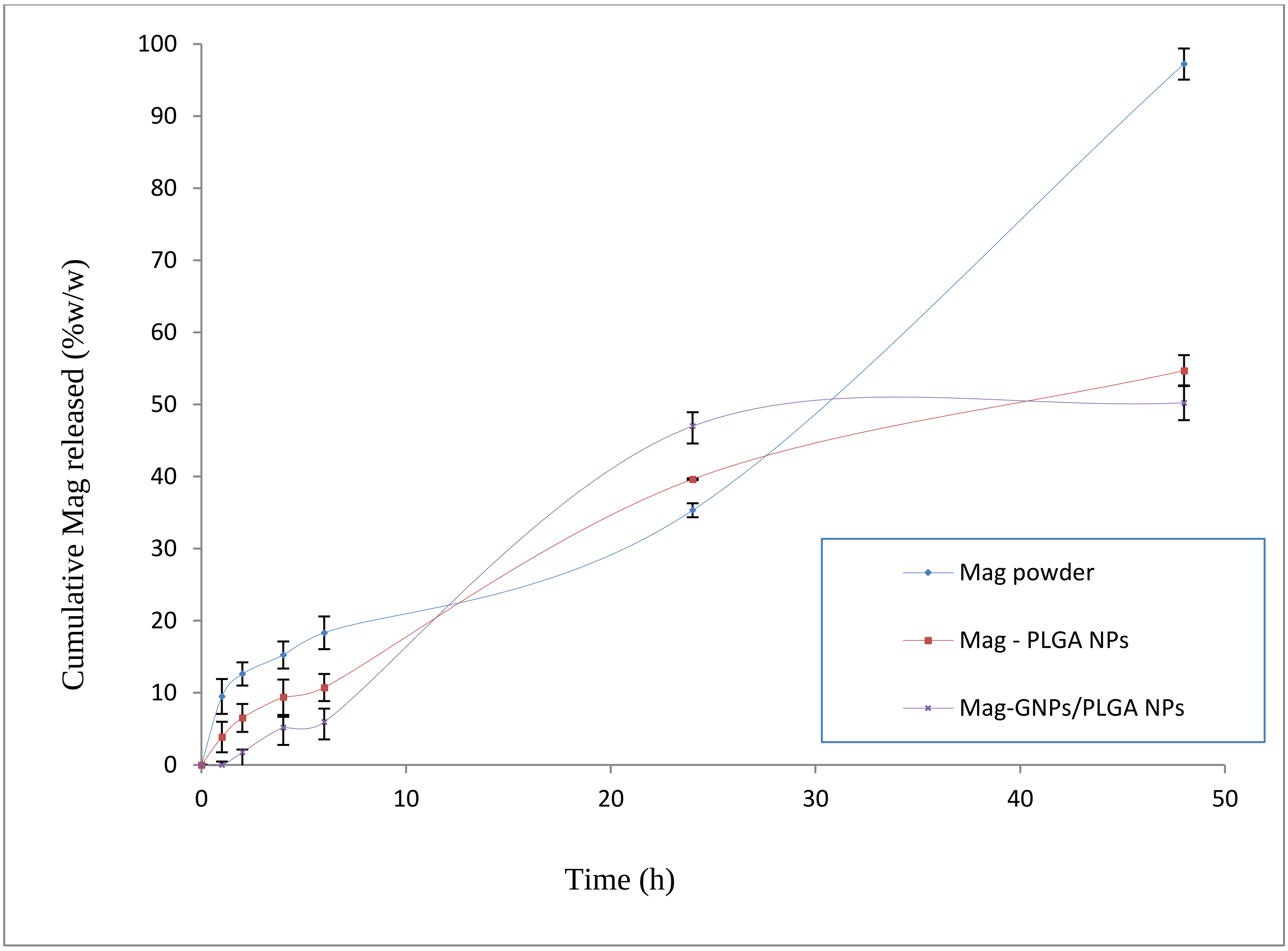

Figure S4 Release profiles of Mag from various formulations in PBS (pH= 7.4) containing 0.5%w/v SDS at 37°C. Presented data are mean of 3 replicates (±standard deviation).

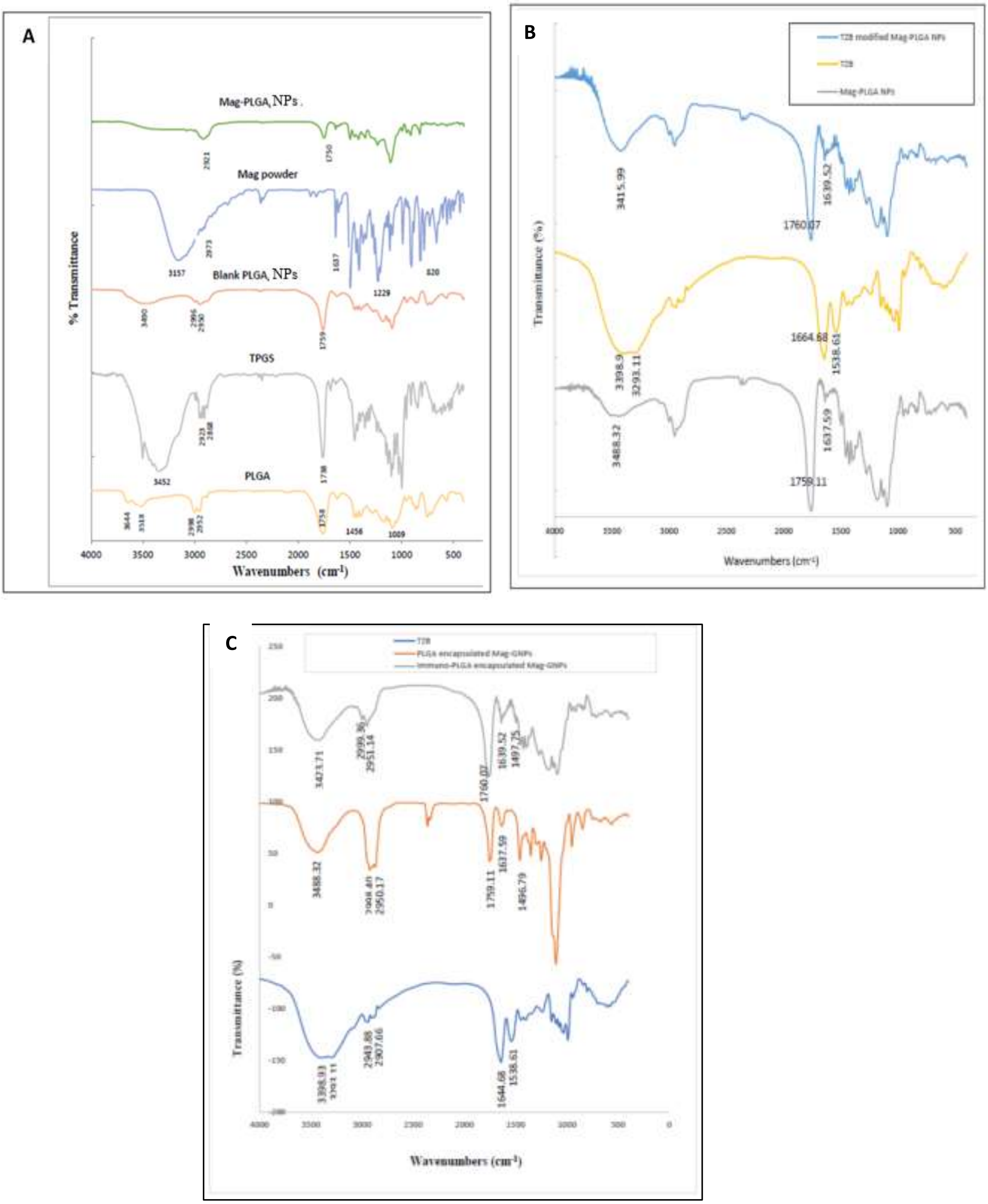

Figure S5 FTIR spectra of (A) PLGA, Mag, TPGS, Blank PLGA NPs, Mag-PLGA NPs; (B) TZB, Mag-PLGA NPs, TZB modified Mag-PLGA NPs and (C) TZB, PLGA encapsulated Mag-Mag-GNPs/PLGA NPs, Mag-GNPs/PLGA-TZB NPs. All PLGA NPs formulae contain TPGS.

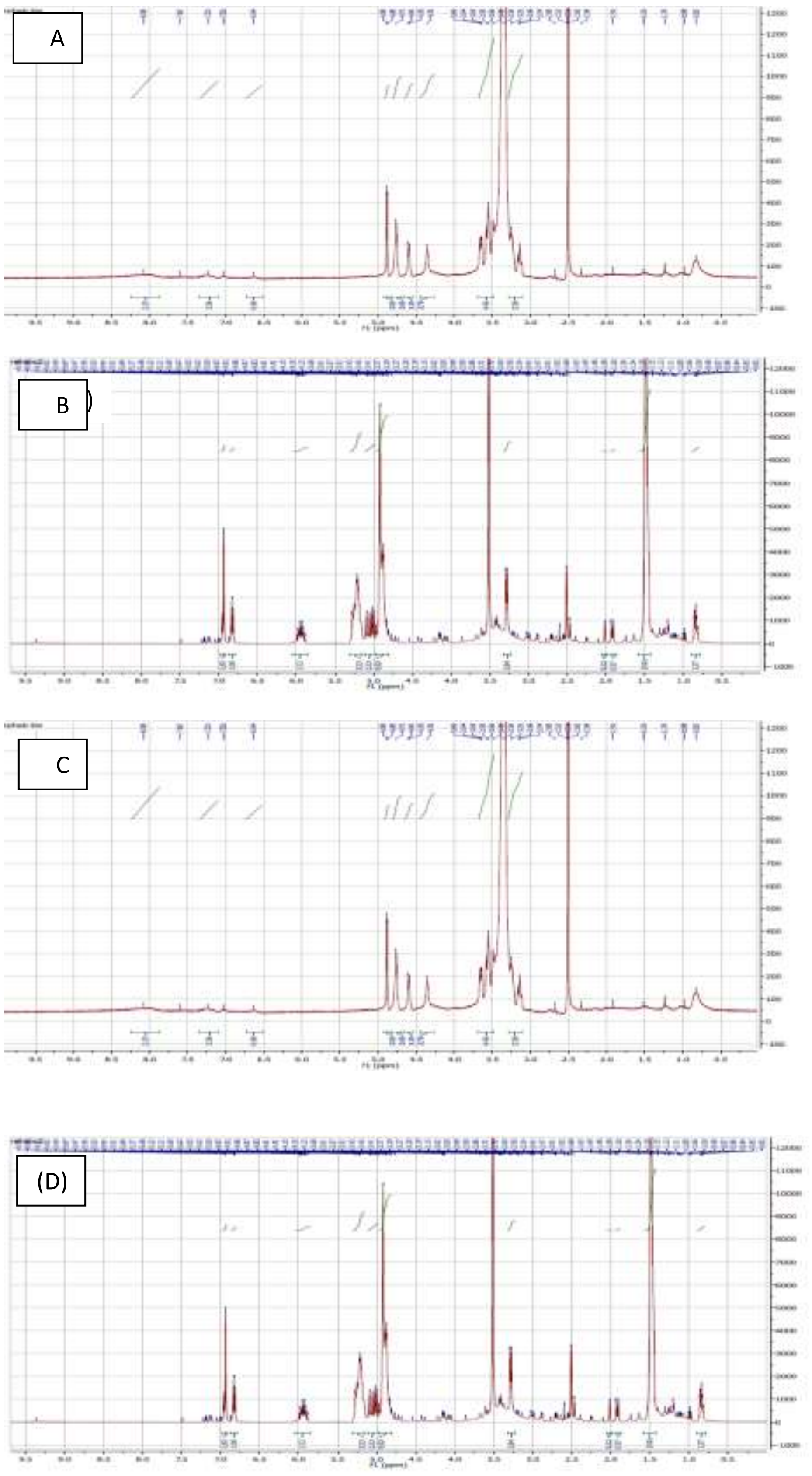

Figure S6 ^{1}HNMR spectra of (A): TPGS, (B) Mag-PLGA NPs, (C) TZB and (D) Mag-PLGA-TZB NPs.

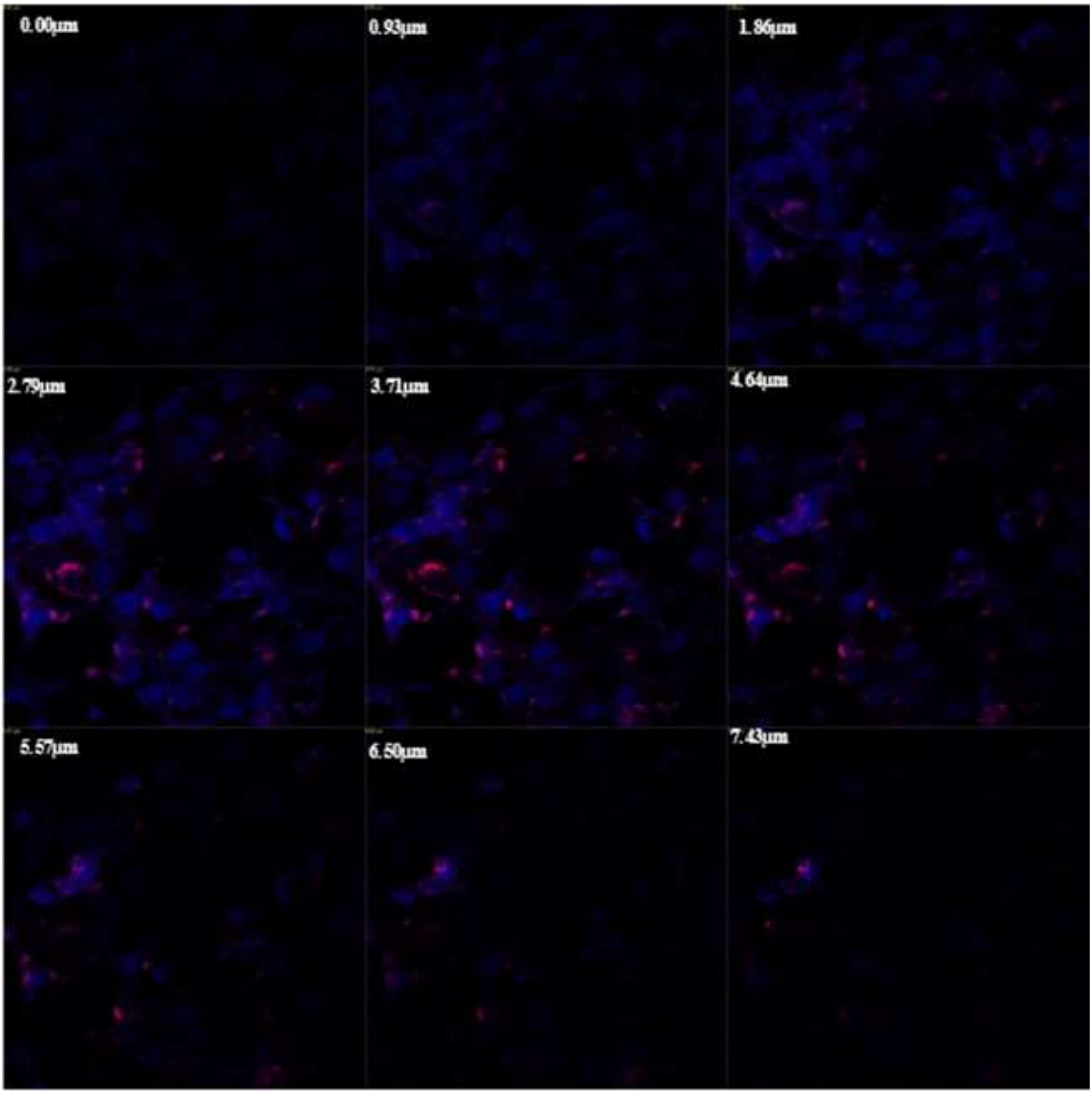

Figure S7 Z-staked CLSM images of MCF-7 cells treated with Nile red-GNPs/PLGANPs. The nuclei were stained with DAPI and the NPs were labeled with Nile red.

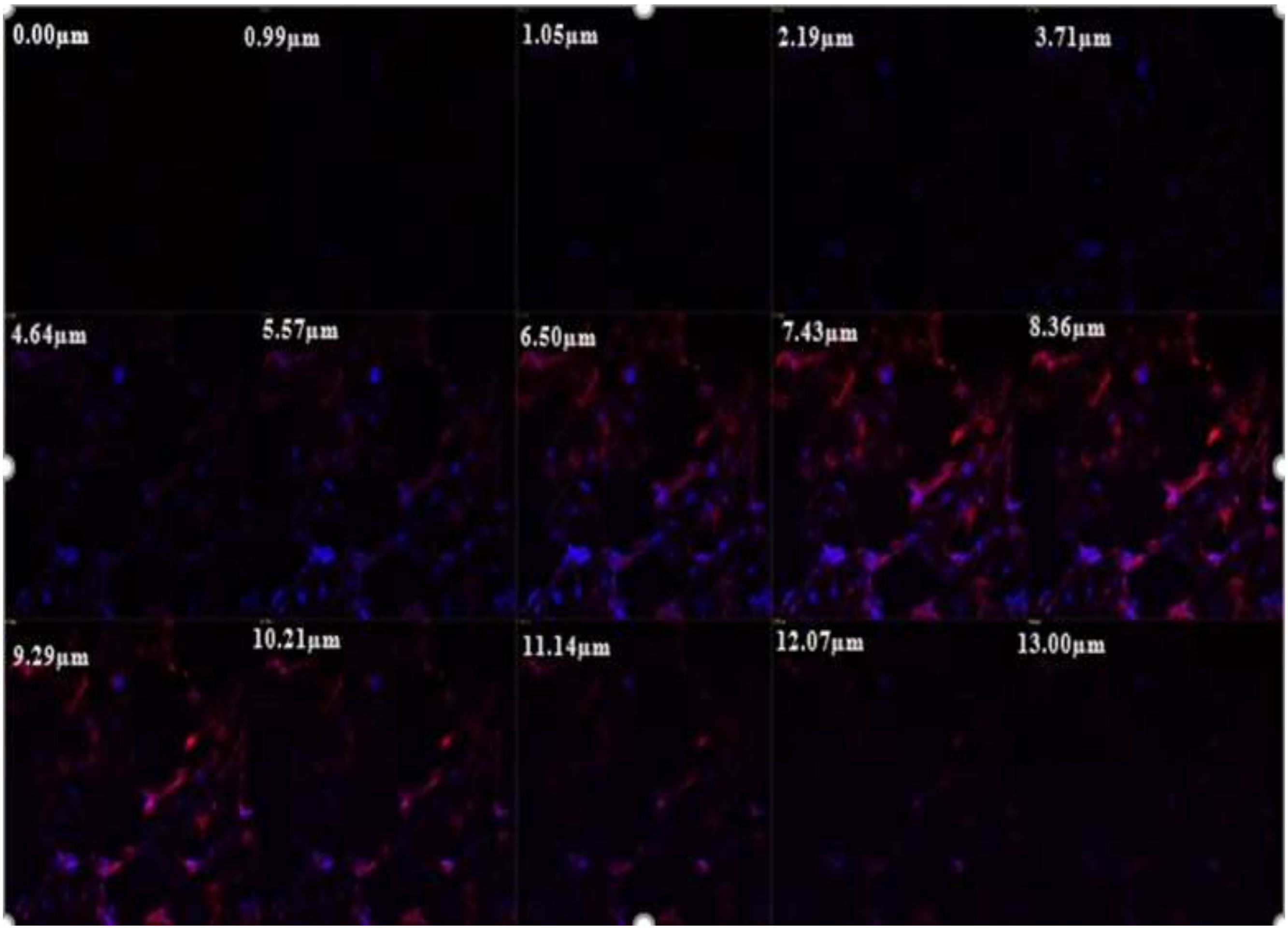

Figure S8 Z-staked CLSM images of MCF-7 cells treated with TZB modified Nile red/GNps-PLGA NPs for 60min. The nuclei were stained with DAPI and the NPs were labeled with Nile red.
